# Unraveling the Role of the Human Gut Microbiome in Health and Diseases

**DOI:** 10.3390/microorganisms12112333

**Published:** 2024-11-15

**Authors:** Mohamad Khalil, Agostino Di Ciaula, Laura Mahdi, Nour Jaber, Domenica Maria Di Palo, Annarita Graziani, Gyorgy Baffy, Piero Portincasa

**Affiliations:** 1 Clinica Medica “A. Murri”, Department of Precision and Regenerative Medicine and Ionian Area (DiMePre-J), Medical School, University of Bari Aldo Moro, 70124 Bari, Italy; mohamad.khalil@uniba.it (M.K.); agodiciaula@gmail.com (A.D.C.); laura.mahdi@uniba.it (L.M.); n.jaber1@studenti.uniba.it (N.J.); 2 Division of Hygiene, Department of Interdisciplinary Medicine, University of Bari Aldo Moro, Piazza Giulio Cesare 11, 70124 Bari, Italy; domenicamaria.dipalo@policlinico.ba.it; 3 Institut AllergoSan Pharmazeutische Produkte Forschungs- und Vertriebs GmbH, 8055 Graz, Austria; graziani@allergosan.at; 4 Division of Gastroenterology, Hepatology and Endoscopy, Department of Medicine, Brigham and Women’s Hospital, Harvard Medical School, Boston, MA 02130, USA; gbaffy@mgb.org; 5 Section of Gastroenterology, Department of Medicine, VA Boston Healthcare System, Boston, MA 02130, USA

**Keywords:** gut barrier, gut microbes, intestinal permeability, leaky gut, microbiome

## Abstract

The human gut is a complex ecosystem that supports billions of living species, including bacteria, viruses, archaea, phages, fungi, and unicellular eukaryotes. Bacteria give genes and enzymes for microbial and host-produced compounds, establishing a symbiotic link between the external environment and the host at both the gut and systemic levels. The gut microbiome, which is primarily made up of commensal bacteria, is critical for maintaining the healthy host’s immune system, aiding digestion, synthesizing essential nutrients, and protecting against pathogenic bacteria, as well as influencing endocrine, neural, humoral, and immunological functions and metabolic pathways. Qualitative, quantitative, and/or topographic shifts can alter the gut microbiome, resulting in dysbiosis and microbial dysfunction, which can contribute to a variety of noncommunicable illnesses, including hypertension, cardiovascular disease, obesity, diabetes, inflammatory bowel disease, cancer, and irritable bowel syndrome. While most evidence to date is observational and does not establish direct causation, ongoing clinical trials and advanced genomic techniques are steadily enhancing our understanding of these intricate interactions. This review will explore key aspects of the relationship between gut microbiota, eubiosis, and dysbiosis in human health and disease, highlighting emerging strategies for microbiome engineering as potential therapeutic approaches for various conditions.

## 1. Introduction

The human body can be considered an ecosystem populated by trillions of living microorganisms which include bacteria, viruses, archaea, phages, fungi, and unicellular eukaryotes [1]. Microorganisms live in the preferred environment on both external parts, i.e., skin, eyes, the exposed part under the nails, entry parts such as mouth, nose, and urogenital tract, and breaks in the skin surface, and internal parts, including the lungs, gut, bladder, kidneys, and vagina.

The gut microbiota is the most complicated ecosystem in nature because it supports vast bacterial populations in the intestine and colon, with roughly 10^11^–10^12^ microorganisms/gram of intestinal content, and the majority are anaerobes (95% of the total organisms) [2]. This community of microorganisms has a collection of genes known as the metagenome, which includes the DNA of many microbial species and is enriched with enzymatic proteins that make a wide range of chemicals and metabolites, as well as compounds produced by the host. Overall, microbial communities, the genomic core, and the interaction of microbial and host chemicals build the microbiome that inhabits the human body in a symbiotic interplay with the host.

In the health status, this interaction is mutually beneficial for the host and for the microbial community, and contributes to the homeostasis of several metabolic pathways, the enterocyte barrier, immunity, and inflammation, with potential effects on weight gain or loss, energy balance, obesity-related disorders, intestinal inflammation, and behavioral responses [3,4].

Further effects of the gut microbiome have been reported in cardiovascular diseases (CVDs), inflammatory bowel disease (IBD), metabolic dysfunction-associated steatotic liver disease (MASLD), depression, Parkinson’s disease, hepatocellular carcinoma, alcoholic liver disease (ALD), immune diseases, chronic kidney diseases (CKDs), and cirrhosis [5,6,7,8,9,10].

In this review, we will present an updated understanding of the role of the gut microbiome in health and a non-exhaustive list of diseases that are associated with a shift in the diversity and density of the microbiome.

## 2. What Are the Fundamental Features of the Gut Microbiome?

Microbiome research has grown substantially in recent years, thanks to technological advancements and considerable reductions in analytic costs. Such studies have opened a wealth of data, yielding enormous insight into the nature of microbial communities, including their interactions and consequences [11].

Recent research has revised the microbial-to-human cell ratio to approximately 1:1, countering the previous estimate of 10:1. This change emphasizes the vast size of the human microbiome’s metagenome, which is about 200 times larger than the human genome, and highlights the importance of microbial genes in functions like metabolism and immune responses [12,13].

At least 2000 species remain largely uncultured candidate bacterial species [14]. Bacterial species make up to 60% of the fecal dry mass. Among 12 different phyla, *Firmicutes* (or *Bacillota)* (60%), *Bacteroidetes* (or *Bacteroidota*) (10%), *Actinobacteria* (10%), and *Proteobacteria*, *Fusobacteria*, and *Verrubomicrobia* are the most common ones and make up to 90% of the total microbial population in humans. The *Firmicutes* and *Bacteroidetes* phyla dominate the gut microbiome in healthy subjects [15,16,17,18].

The microbial composition is highly variable and highly personalized among individuals and can be arranged as the core microbiota, which is constantly associated with a given host genotype or a specific environment, or as the transient microbiota, which can change over time [1,4].

Ultimately, the gut microbiome in health depends on local factors such as pH, temperature, oxygen concentration, osmolarity, and pressure. The mucin layer is another key component, a carbon source for microbes, and the site for bacterial adhesion [2,15,19] within the complex multilayer system which includes the microbial species floating within the upper layer of mucin, the enterocyte layer, the immunological layer, the endothelial layer, the liver layer, and the functional apparatus involving gastric, biliary, and pancreatic secretes and intestinal propulsive waves [19].

In addition, external factors include developmental factors, nutrient supply [20], lifestyle (i.e., dietary habits, physical activity [21], and smoking [22,23]), age, and environment, such as geography, climate, systemic disorders, drugs [18,24], toxic chemicals [25,26], air pollution [27], and diseases [28]. Altogether, such conditions contribute to shaping the microbiome profile and the microbial growth in a suitable environment (Table 1).

Developmental factors exist since birth and are due to gestational age, maternal diet and microbiome, and medications. The method of delivery confers different exposure to microorganisms, in that vaginal delivery is followed by the inoculation of the newborn by the mother’s vaginal and fecal microbiota, e.g., *Bifidobacterium* spp., while the Caesarean delivery is followed by the inoculation of skin and environmental microbes [29]. At birth, the gut is colonized by facultative anaerobes which create anaerobic conditions which select obligate anaerobes, i.e., *Bifidobacterium* and *Bacteroides* spp., within 2 weeks [30].

In the infant, factors to be considered are breastfeeding (higher proportions of *Bifidobacteria*, more stable and less diverse bacterial community) vs. formula milk feeding, liquid vs. solid food (more *Firmicutes)* intake, geographical location, medications, hygiene, the general features of family members, the host health, and maternal diet [31]. In general, by the age of 3, the gut microbiome profile is similar to the adult microbiome [32]. In toddlers, childhood, and adults, the microbiome is further shaped by the host’s hygiene and health, lifestyle, and medications [15]. Thus, after early life maturation, the fecal microbiome in each adult individual is fairly stable over time [33]. Despite this, in the elderly, the microbiota diversity decreases for *Bifidobacteria* and increases for *Enterobacteriaceae*. In parallel [34,35]. Likewise, the abundance of *Bacteroidetes* increases, whereas the *Firmicutes* phyla becomes less abundant at >65 years [36].

Eventually, the adult-type microbiome becomes a complex multi-stable ecosystem [37] characterized by phylogenetic diversity among different gut segments and individuals and involving dominant and subdominant taxa. Essential ecological features are stability, resistance, resilience [38], diversity, abundance, and functional redundancy, i.e., similar functions are performed by different bacteria [39]. Such features develop within symbiotic interactions with the body host and contribute to health-related aspects of nutrient metabolism, signaling to immune cells to keep physiological balance and immunity, and defend against colonization by pathogenic and opportunistic microbes [40,41,42]. This latter function includes the maintenance of intestinal epithelium integrity [43].

Indeed, the close relationship between the microbiome and the host may be beneficial, pathogenic, or neutral [4], with the microbiome having a significant impact on human health and disease [44,45,46] and contributing to the regulation of host physiology by the activation of several enzymatic proteins and metabolites able to affect host metabolism and several pathways [47,48]. Certainly, human health relies on eubiosis, a condition that implies a preserved gut barrier, i.e., the complex and highly interactive morpho-functional system where the microbiome, floating in the outer mucin layer, has crosstalk with enterocytes, secretions, immune cells, endothelium, and the portal–liver barrier [19,49].

The protective role of the microbiome starts in the oral cavity, i.e., when the resident microbial community reduces nitrate to nitrite, which is then converted to nitric oxide with an antimicrobial effect, protecting the vasculature [50]. The *Streptococcus salivarius* strain K12 creates an environment enriched in bacteriocin that is unfavorable to the colonization of pathogenic Gram-negative species associated with periodontitis [51].

**Figure 1:**
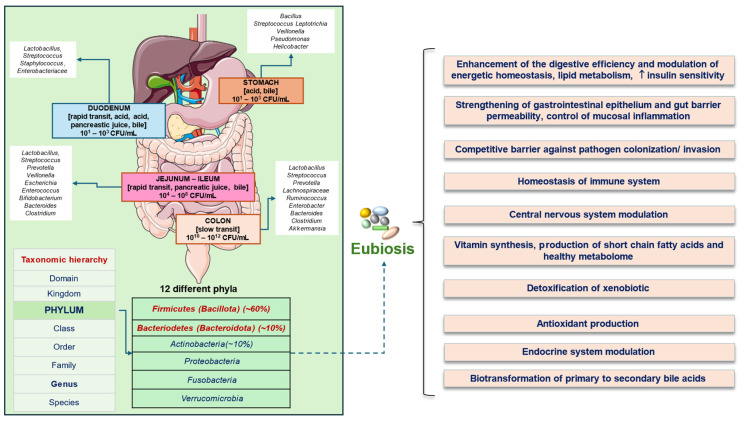
Density, type, and distribution of microbiome in the human gastrointestinal tract. The prevalent phyla of the human gut are depicted, along with the most represented genera which populate different gut segments. A vast majority of commensal bacteria are found in the colon. A lower bacterial population is found in the stomach and small intestine. Activation of specific gut–organ/apparatus axes in health is indicated. Legend: CFU, colony forming unit, ↑: increased.

Figure 1 depicts the most prevalent phyla distributed in the gut and genera at different levels, along with some beneficial functions of gut microbiome in health.

In the stomach, the microbial community is influenced by acidic gastric juice, bile reflux, oral microbiota, and mucin thickness. The most represented phyla in gastric juice are, *Actinobacteria*, *Bacteroidetes*, and *Fusobacteria*, while *Proteobacteria* and *Firmicutes* prevalently populate the gastric mucosa [4]. Notably, infection by the species *H. Pylori* makes this microorganism the most predominant bacterium in the stomach and is followed by the alteration of the gastric microbiome community [52,53].

In the small intestine, the transit time is about 3–5 h and the presence of bile acids in bile has antimicrobial activity [54,55,56,57]. At this level, the community is mainly composed of facultative anaerobes, including phyla *Proteobacteria* and *Bacteroides* populated by *Streptococci*, *Lactobacilli*, and *Enterococci* species [58,59].

The colon has a slow transit time, has a mildly acidic to neutral pH level, and is mostly populated by obligate anaerobes. The predominant phyla are *Firmicutes* and *Bacteroides* [60], which include, among *Firmicutes*, *Ruminococcus* spp., but also potentially harmful species, i.e., *Staphylococcus aureus* and *Clostridium perfringens.* In addition, with the phylum *Proteobacteria*, well-known pathogens include *Enterobacter*, *Helicobacter*, *Shigella*, *Salmonella*, and *Escherichia coli.* Among the *Verrucomicrobiota* phylum the anaerobic Gram-negative *Akkermansia muciniphila* spp. colonizes the mucin layer with mucin-degrading properties.

Disease-related variations may lead to profound disruption of the gut microbiome profile [61], a condition named dysbiosis, i.e., a shift of microbiota, and will increase the chance of systemic alterations [62] linking the unbalanced microbiome with several non-communicable diseases such as hypertension, obesity, cardiovascular disorders, diabetes, and IBD [63,64,65]. Dysbiosis is also a predisposing condition for the emergence and outbreak of pathogens [66,67]. Distinct profiles of the microbiome are often associated with specific diseases when the comparison is made between healthy individuals. Notably, dysbiosis can occur at any level of the taxonomic rank, i.e., from phylum- to species-level [68].

Intake of antibiotics, especially in early life during prenatal, intrapartum, and postnatal periods, can decrease microbiome diversity. Long-lasting effects can include increased risk of very early-onset IBD and overweight/obesity. Rasmussen et al. [69] conducted a systematic review and meta-analysis of observational studies investigating the association between antibiotic exposure in infancy and the risk of childhood overweight and obesity. A total of 527,504 children were included in 13 studies. Antibiotic exposure in infancy increased the odds ratio (OR) of childhood overweight and obesity (OR 1.11, 95% confidence interval [CI] 1.02–1.20). Particularly, exposure to >1 treatment was associated with an OR of 1.24 (95% CI 1.09–1.43), and exposure within the first 6 months of life was associated with an OR of 1.20 (95% CI 1.04–1.37). This association can imply the direct effect of antibiotics on the gut microbiome.

On the whole, microbiome research has grown significantly due to technological advancements, providing detailed insights into microbial communities and their impact on health. Early-life factors like maternal diet, delivery mode, and feeding type influence initial microbiome composition, with a stable adult-like microbiome typically established by age three. Adult microbiomes are shaped by diet, lifestyle, and environmental exposures, remaining stable but showing diversity shifts in older age. Microbiome composition varies by body region, with acidic environments favoring specific bacteria and colon-supporting anaerobes. Disruptions, or dysbiosis, are linked to diseases like obesity, diabetes, and cardiovascular disorders. The gut microbiome’s vast metagenome, larger than the human genome, plays essential roles in metabolism, immunity, and protection against pathogens.

## 3. To What Extent Is Human Metabolism Influenced by the Gut Microbiome?

The enzymatic patrimony of the gut microbiome is involved in several pathways and contributes to the generation of metabolites directly from diet, biotransformation of products produced by the host, or ex novo. Such metabolites contribute to some key functions for the host in health. In addition, several metabolites have been associated with systemic diseases, depending on environmental and host factors (Table 2). The most extensively studied metabolites are short-chain fatty acids (SCFAs), bile acids (BAs), and trimethylamine-N-oxide (TMAO) [70,71].

**Table 2:** Major metabolites produced by the gut microbiome.

Class	Metabolite(s)	Target(s)	Associated Functions	Potentially Associated Diseases	References
**Short-chain fatty acids (SCFAs)**	Acetate Propionate Butyrate Hexanoate Isovalerate Isobutyrate 2-methyl propionate Valerate	Receptors GPR41 GPR43 GPR109A GPR81 GPR91 HDAC1 HDAC3	Profiling of gut microbiota composition Maintaining gut barrier integrity Energy homeostasis Production of gut hormone Control of appetite Modulation of immune system Anti-inflammatory inhibition of proinflammatory cytokines Control of circadian clocks Modulation of water and sodium absorption	Obesity Diabetes MASLD Hypertension, atherosclerosis Metabolic syndrome Pancreatitis Inflammatory bowel diseases Chronic kidney disease Radiation proctitis Diarrhea Colorectal cancer Autism Parkinson’s disease	[72,73,74,75,76,77]
**Bile acids**	Secondary BAs Cholic acid Deoxycholic acid Lithocholic acid Tertiary BAs Ursodeoxycholic acid	Nuclear receptors, FXR, VDR, PXR/SXR Constitutive androstane receptor GPBAR-1 Membrane-associated receptor GPBAR-1 Sphingosine 1-phosphate receptor 2 (S1PR2) Formyl-peptide receptor (FPR) Muscarinic acetylcholine receptor (mAChR)	Regulation of fat and fat-soluble vitamin absorption Modulation of gut microbiota Modulation of gut hormones and motility Regulation of the immune system, digestion, gluco-lipid, amino acid homeostasis Regulation of neurotransmission Control of circadian clocks	Primary biliary cholangitis Primary sclerosing cholangitis Bile acid malabsorption-diarrhea Obesity atherosclerosis Metabolic dysfunction-associated steatotic liver disease Metabolic dysfunction-associated steatohepatitis Inflammatory bowel diseases Hepatic encephalopathy Parkinson’s disease Alzheimer’s disease Traumatic brain injury Multiple sclerosis Stroke Cancer	[78,79,80,81,82,83]
**Tryptophan and indole derivatives**	Indole Serotonin Indole-3-propionic acid Indole-3-lactic acid Indole acetic acid Indole-3-acetamide Indole pyruvic acid Indoxyl sulfuric acid	AhR PXR	Regulation of gut barrier, hormone, and motility Modulation of the immune system Microbial spore and biofilm formation	Irritable bowel syndrome Inflammatory bowel diseases Mucosal candidiasis Obesity Stroke Parkinson’s disease Alzheimer’s disease Autism Schizophrenia	[84,85,86,87,88]
**Microbial toxins**	Lipopolysaccharide (LPS), peptidoglycan (PGN), lipoteichoic acid (LTA)	TLR4	Promotion of local and systemic inflammation	Obesity atherosclerosis Metabolic dysfunction-associated steatotic liver disease Metabolic dysfunction-associated steatohepatitis	[89,90,91]
**Gases**	H_2_S H_2_ NO CO_2_ CH_4_	H_2_S → sulfhydration of target proteins NO → soluble guanylate cyclase	H_2_S: Modulation of gut inflammation and motility. Regulation of epithelial secretion, and susceptibility to infections NO: Modulation of gastric mucosal protection CH_4_: Modulation of gut motility	Colitis Ulcer Parkinson’s disease	[92,93,94,95]
**Choline metabolites**	Choline TMAO Betaine	Activation of NF-κB Protein kinase C (PKC) NLRP3 inflammasome	Increasing inflammation Mitochondrial dysfunction Thrombosis Promoting myocardial hypertrophy Inhibition of bile acid synthesis	Metabolic dysfunction-associated steatotic liver Atherosclerosis disease Obesity Hypertension Heart failure	[96,97,98]
**Vitamins**	Thiamine (B1) Riboflavin (B2) Niacin (B3) Pyridoxine (B6) Pantothenic acid (B5) biotin (B7) Folate (B11-B9) cobalamin (B12) Menaquinone (K2)	Vitamin receptors	Provision of vitamins for hosts Cellular and sub-cellular function modulation of immune function Cellular metabolism and survival	Dementia Schizophrenia Autism	[99,100]
**Neurotransmitters**	GABA Dopamine 5-HT Catecholamines	GABA receptors Adrenergic receptors 5-HT receptors	Gut motility Stress responses Regulation of the immune system Function of the nervous system	Autism Parkinson’s disease	[101,102,103]
**Others**	Ethanol Polyamines (putrescine, spermidine, and spermine) Phenolic derivatives (4-OH phenylacetic acid, urolithins, enterodiol, and 9-prenylnaringenin) Triphosadenine Organic acids (benzoate and hippurate)	Triphosadenine activate P2X and P2Y receptors	Modulation of gut barrier and systemic immune response and inflammation Profiling gut microbiota composition	Inflammatory bowel disease, irritable bowel syndrome Metabolic dysfunction-associated steatotic liver *C. difficile* and *H. pylori* infections	[104,105,106,107]

Abbreviations: AhR, aryl hydrocarbon receptor; BAs, bile acids; CH_4_, methane; GPR, protein-coupled receptors (GPRs); HDAC, nuclear class I histone deacetylases; H_2_S, hydrogen sulfide; MASLD, metabolic dysfunction-associated steatotic liver disease; TMAO, trimethylamine-N-oxide.

The colonic microbiome is continuously processing host-derived substrates, i.e., mucus, pancreatic enzymes, bile acids, and exfoliated epithelial cells. The gut microbes, especially in the colon, come into contact with and process dietary components escaping digestion in the upper gastrointestinal tract to bioactive food components. Diet is a feasible and easy tool to maintain homeostasis (eubiosis) or increase the diversity of the gut microbiome [77,108,109,110,111,112,113], although it is largely unknown what type of diet will ultimately lead to a healthy and stable microbiome [70]. In general, diet can influence the microbiome, and gut microbes can shape the biochemical metabolomic profile of the diet [114]. By contrast, the host immune system and several diseases, e.g., obesity and diabetes, can interact with the gut microbiome [112,115,116,117,118,119] and, in turn, can modulate several intestinal inflammatory outcomes [115,116,120]. More environmental factors can also influence the gut microbiome, including helminth exposure [121] and the use of antibiotics [69], but also exposure to ingested toxic chemicals [122,123] and air pollution [122] (see specific paragraph).

The role of the gut microbiome in contributing to host energy harvesting and metabolic efficiency is a matter of active research [124], since the bacterial metabolic output depends on the availability of substrates [125,126].

The two main types of colonic microbial fermentation are the saccharolytic fermentation of carbohydrates (mainly in the proximal colon) with the production of SCFAs and lactate, and the proteolytic fermentation of protein with the production of amino acids, branched-chain fatty acids, small amounts of SCFAs, and potentially harmful metabolites, such as phenols, sulfides, ammonia, amines, and indoles [127].

The production of branched-chain fatty acids (BCFAs) including isobutyrate and isovalerate has an effect on histone deacetylase (HDAC) inhibition and increased histone acetylation [128].

**Figure 2:**
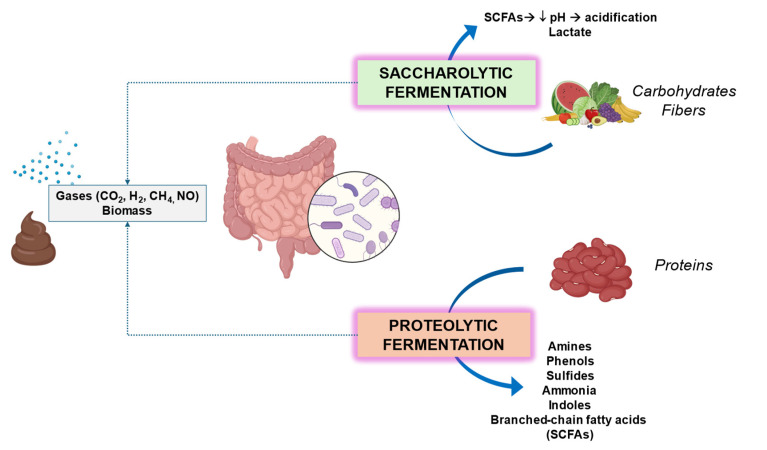
Metabolites are produced during the colonic fermentation of carbohydrates and proteins. Legend: CO_2_, carbon dioxide; CH_4_, methane; H_2_, hydrogen; NO, nitric oxide; SCFAs, short-chain fatty acids. Created with Biorender.com.

Both saccharolytic and proteolytic fermentations contribute to the production of gases such as microbiome-derived compounds and those functional to the host, including gases such as hydrogen (H_2_), methane (CH_4_), carbon dioxide (CO_2_), hydrogen sulfide (H_2_S), and nitric oxide (NO) [39] (Figure 2).

In general, carbohydrates contribute as energy sources and as fermentative substrates to produce key biomolecules for the host [129]. By contrast, if carbohydrates are not sufficient, bacteria will switch to an alternative energy source, i.e., proteolytic, with metabolites potentially harmful to human health [24].

Saturated aliphatic acids with carbon chains ranging from 1 to 6 atoms are known as SCFAs. They originate from the saccharolytic fermentation of microbiome-accessible carbohydrates (MACs), i.e., complex undigested carbohydrates in dietary fiber such as cellulose, hemicelluloses, resistant starch, pectin, oligosaccharides, and lignin. A minor aliquot of SCFAs can originate from undigested proteins and peptides containing branched-chain amino acids (BCAAs) which are metabolized to branched-chain fatty acids (i.e., 2-methyl butyrate and iso-valerate) [102]. The major SCFAs are acetate, propionate, and butyrate, found in human feces in a 3:1:1 to 10:2:1 molar ratio, consistent with the values reported in the intestine in early sudden deaths [24] (Table 2). Acting both locally in enterocytes and systemically throughout the body, SCFAs influence a range of physiological processes [130,131,132,133] since they can be transported into the circulation system and other tissues such as the brain, heart, and lungs [77,134]. Butyrate is the primary source of energy for human colonocytes [77,135], and has anti-inflammatory and anti-carcinogen properties [39,77] via the apoptosis of colon cancer cells and regulation of gene expression by inhibiting histone deacetylase [136,137]. Propionate provides an energy source for the epithelial cells, while in the liver, it plays a vital role in gluconeogenesis [138]. Acetate, as a co-factor, helps the growth of other bacteria, e.g., *Faecalibacterium prausnitzii*, which will not grow in pure culture in the absence of acetate [24]. Butyrate has additional systemic effects and the effects of SCFAs are mainly mediated by specific action on protein-coupled receptors (GPRs) (especially 41 and 43) expressed on the pancreas, liver, adipocytes, brain, enteroendocrine L-cells, and immune cells, which explain the effects on appetite (via stimulation of peptide YY (PYY), leptin, and glucagon-like peptide-1 (GLP-1), and attenuation of insulin resistance), energy harvest, and hepatic lipid metabolism [39,77]. These effects contribute to the maintenance of body weight [139,140]. Interacting with class I nuclear histone deacetylases (HDACs), such as HDAC1 and HDAC3, SCFAs influence a range of cellular processes. These enzymes, widely expressed across cell types, play crucial roles in cell survival, aging, and the promotion of anti-inflammatory phenotypes. In general, a diet enriched in fiber will maintain the diversity of the intestinal microbiome and will involve SCFA production [141]. By contrast, many diseases have been associated with the defective functionality of the fiber–microbiome–SCFA–receptor axis (Table 2).

The colonic microbiome will also govern the biotransformation of colonic BAs. These BAs are cholesterol-derived amphipathic and water-soluble metabolites secreted in human bile as primary BAs (i.e., cholic acid and chenodeoxycholic acid) and transformed by colonic microbiome to secondary BAs (deoxycholic acid, lithocholic acid), and tertiary BAs (ursodeoxycholic acid) [2,15,77,109,142,143]. Upon hepatic synthesis or recirculation, BAs are conjugated to taurine or glycine to increase their solubility [144]. During fasting, BAs are excreted in bile and concentrated in the gallbladder. After meal/fat-induced stimulation, BAs are discharged into the duodenum during gallbladder contraction. Following the intestinal progression, most (>95%) of the primary BAs are actively reabsorbed in the terminal ileum and enter the portal vein to re-enter the liver via active uptake, a process termed enterohepatic circulation, to maintain the bile acid pool. A small number of primary BAs escape ileal reabsorption, enter the colon, and are available for microbiome biotransformation to secondary and tertiary Bas, mainly via bile salt hydrolases and 7*α*-dehydroxylases. Most secondary/tertiary BAs will undergo passive colonic reabsorption and will enter (unconjugated) the enterohepatic circulation [56,79,80]. Key to the process of biliary cholesterol excretion are micelles and phospholipid-enriched vesicles, which are formed and function under the influence of BAs. Digestion of dietary fat also relies on the micellization of lipids at the enterocyte level, especially in the small intestine. In the terminal ileum and before their active reabsorption, BAs are sensed by specific L-type enterocytes and their receptors in the terminal ileum, i.e., the membrane-associated G protein-coupled bile acid receptor 1 (GPBAR-1), leading to release of the enterokines GLP-1/2 and PYY. The effect on the nuclear receptor farnesoid X receptor (FXR) will release the enterokine FGF-19 in humans [143]. Other nuclear receptors include the vitamin D3 receptor (VDR), pregnane X receptor/steroid and xenobiotic-sensing receptor (PXR/SXR), constitutive androstane receptor, sphingosine 1-phosphate receptor 2, formyl-peptide receptor, and muscarinic acetylcholine receptor [78]. By such complex cross-talks, BAs regulate their synthesis, cholesterol, lipid, glucose, energy metabolism, and several other functions, including anti-inflammatory and antimicrobial effects [145]. Several diseases have been associated with the dysfunction of BA pathways (Table 2).

As previously mentioned, proteins and peptides escaping host digestion can be metabolized by the gut microbiome in additional metabolites which include ammonia, amines, sulfides, nitrogen compounds, indoles, phenols, p-cresol sulfate, and precursors to branched-chain fatty acids [146]. Unabsorbed tryptophan can become the substrate for the microbiome to tryptamine, skatole, indole, and indole derivatives such as indoxyl sulfate, indole-3-propionic acid, 3-methyl-indole, and [96]. The receptor aryl hydrocarbon receptor (AhR) can influence metabolism, immunity, and social behavior in hosts [85,86].

Indole derivatives include indole-3-propionic acid (IPA) with neuroprotective and antioxidant effects, with effects on the regulation of gut barrier function. Indoxyl sulfate is a uremic toxin which can accumulate in the blood of individuals with impaired excretion systems [147].

Phenolic derivatives include 4-OH phenylacetic acid, urolithins, enterodiol, and 9-prenylnaringenin. These molecules have antimicrobial effects, maintain intestinal health, and protect against oxidative stress [148].

Polyamines include putrescine, spermidine, and spermine which contribute to the high proliferation rate of intestinal epithelial cells. They also enhance the gut barrier integrity and the adaptive immune system [149,150].

Dietary amino acids choline, carnitine, and phosphatidylcholine are abundant in red meat, fish, and eggs and can be sequentially metabolized by both gut microbiomes and hosts. Carnitine is metabolized to trimethylamine (TMA) in the gut lumen and converted in the liver by flavin-containing monooxygenases 1 and 3 into TMAO. Increased serum TMAO levels are associated with an increased risk of cardiovascular diseases through the promotion of atherosclerotic lesions via the regulation of lipid metabolism, and glucose synthesis [151], though this aspect remains controversial [152].

Other amino acid metabolites act as neurotransmitters since microbes can synthesize the phenylalanine and tyrosine derivative dopamine via decarboxylation of L-DOPA by tyrosine decarboxylase [153]. A further step is the conversion of dopamine to norepinephrine (hydroxylation) and epinephrine (methylation). The gut microbiome is also able to synthesize neurotransmitters like serotonin (5-hydroxytryptamine (5-HT)), norepinephrine, and γ-aminobutyric acid (GABA) [154].

The gut microbiome synthesizes lipopolysaccharides (LPSs), conjugated fatty acids, and other lipids that modulate host functions [105]. Lipopolysaccharide (LPS), peptidoglycan (PGN), and lipoteichoic acid (LTA) are characterized by epigenetic regulation of genes in colorectal cancer, modulation of chromatin structure, and transcriptional activity [128,155].

Genomic studies find that about half of bacteria possess pathways for B-vitamin biosynthesis, another important function for the host [156]. Few vitamins can be synthesized by the microbiome such as biotin, folate, and vitamin K, and neutralize carcinogenic compounds, such as pyrolysates [157]. Microbial metabolites can bind to specific host membranes or nuclear receptors [158]. Vitamins include thiamine (B1), riboflavin (B2), niacin (B3), pyridoxine (B6), pantothenic acid (B5), biotin (B7), folate (B11-B9), cobalamin (B12), and menaquinone (K2), which contribute to red blood cell formation, DNA replication, and repair, work as enzymatic co-factors, and enhance immune functioning [105,159].

Ethanol and protein fermentation metabolites may be involved in NAFLD progression [160,161]. The production of hydrogen sulfide (H_2_S) can reduce/neutralize the reactive oxygen species [128,162]. Microbes also play a role in xenobiotic metabolism with changes in the chemical structures of drugs, pollutants, several pesticides [163], and plant polyphenols [24]. Other microbiome-derived compounds functional to the host include gases (Figure 2) such as hydrogen (H_2_), methane (CH_4_), carbon dioxide (CO_2_), hydrogen sulfide (H_2_S), and nitric oxide (NO), which can influence host physiology [164]. Nevertheless, most of the metabolites deriving from the gut microbiome remain to be investigated and functionally characterized. Metagenomic, sequence-based (meta)genomic, and metabolomic studies will be required shortly [165]. Foods and several food components can induce transient changes in the gut microbiome composition [166]. Some foods like fish, meat, and fiber can produce long-term effects [167].

In addition, the resilient microbiome can adapt to major dietary changes and changes are as fast as 1–2 days when individuals change their dietary pattern from plant to meat-based diets. Changes also take place when increasing the daily intake of dietary fiber to more than 30 g or adopting either a high-fiber–low-fat diet or a low-fiber–high-fat diet for ten days [110,168]. Factors involved in such resilient response can involve daily changes in dietary patterns, the need for energy production, and huge variability of the microbial community which is involved in the degradation of proteins, dietary fibers, and peptides by fermentation and anaerobic degradation [169]. More in detail, the phyla *Bacteroidetes* and *Firmicutes* ferment fibers [170,171]. On the other hand, dietary unsaturated plant-based fats inhibit detrimental bacteria and increase the abundance of beneficial *Bifidobacterium* and butyrate-producing bacteria (*Roseburia* and *Faecalibacterium*) [172]. Hoffmann et al. [173] investigated the associations of diet with fungal and archaeal populations in well-characterized individuals. For fungi, 66 genera were characterized with the generally mutually exclusive presence of either the phyla *Ascomycota* or *Basiodiomycota*. For archaea, *Methanobrevibacter* was the most prevalent genus (30% of samples). Diet was associated with several expressions of fungi and archaea, with each other, and with bacterial lineages. In particular, *Methanobrevibacter* and *Candida* were positively associated with carbohydrate-enriched diets, but negatively with diets high in amino acids, protein, and fatty acids. *Candida* abundance was most particularly associated with the recent consumption of carbohydrates, while *Methanobrevibacter* abundance was associated with both long-term and recent consumption of carbohydrates. Micronutrients can further contribute to changes in the gut microbiome and beneficial health effects [174].

In short, the gut microbiome is a vital part of the body, producing essential metabolites from various sources such as dietary inputs, host-derived compounds, and de novo synthesis. These metabolites, such as SCFAs, BAs, and TMAO, are essential for health but may also contribute to systemic diseases influenced by diet, immune interactions, and environmental factors. Diet plays a crucial role in maintaining microbiome balance, but the exact impact on microbial stability remains unclear. Colonic microbes ferment carbohydrates, producing SCFAs and proteins while releasing gases beneficial for host functions. The microbiome also converts bile acids, aiding fat digestion and metabolic regulation. Diet significantly influences microbiome composition, with fiber and plant-based fats supporting beneficial bacteria and resilience to dietary shifts maintaining stability.

## 4. To What Extent Do External Factors Linked with Environmental Pollution Influence the Microbiome?

The gut microbiota represents the first interface between the external environment and the human body at the level of the intestinal barrier. Thus, toxic chemicals contaminating ingested food and water can play a remarkable role in the modulation of the gut microbiota, potentially generating gut dysbiosis. Chronic exposure to toxic chemicals such as cadmium, chromium, lead, nickel, bisphenol A, phenanthrene, triclosan, polychlorinated biphenyls, and pesticides can affect the gut microbiota diversity and relative abundance of specific microbial species [175,176,177,178,179,180,181,182,183,184,185,186,187,188,189,190,191,192,193,194]. As a result, exposure to toxic contaminants alters the gut barrier and the production of microbial metabolites, leading to pathogenic effects both locally and in distant organs such as the liver, brain, and cardiovascular system. Exposure to metals such as cadmium [184] and lead [195], to endocrine-disrupting chemicals such as bisphenol A [196], phthalates [197], triclosan [188] or to persistent organic pollutants such as polychlorinated biphenyls, polybrominated diphenyl ethers [198] or pesticides [199,200,201,202] generate gut dysbiosis which, in turn, can predispose the host to altered metabolic homeostasis, insulin resistance, and obesity.

Emerging evidence from animal [203,204,205] and human studies [206,207] also suggests a critical role for the ingestion of microplastics, widespread contaminants of environmental matrices, and food able to induce gut dysbiosis and the altered production of microbial metabolites.

Accumulating evidence also points to air pollutants as strong modulators of the gut microbiome, and to their ability to generate gut dysbiosis from infancy through to old age [27]. Air pollutants can reach the gastrointestinal tract through systemic circulation (fine particles and gaseous pollutants crossing the lung filter) but also due to particle swallowing following mucociliary clearance (larger particles). Epidemiological studies suggest that the diversity of the gut microbiome is negatively associated with particulate air pollution, with the increase in taxa belonging to *Bacteroidetes*, *Deferribacterota*, and *Proteobacteria*, and decreased *Verrucomicrobiota* [27]. On the other hand, evidence from animal and experimental studies has documented that air pollutants can promote gut damage, local inflammation, oxidative stress, and increased gut permeability [27].

## 5. Is Immune Homeostasis Also Governed by the Gut Microbiome?

Proper and early development of the gut microbiome has a role in immune function and prevents autoimmune disorders [208,209,210,211]. The gut microbiome stimulates and contributes to the maturation of the immune system in response to pathogens, and this step is important to induce and sustain tolerance [212]. The maturation of the immune system begins at birth in the presence of commensal microbial species, and both adaptive and innate immune responses are partly regulated during maturation by the gut microbiome. Germ-free animal models show that the gut microbiome is essential in this respect [213].

Innate immunity is widely expressed with the gut barrier, as a layer of cells below the physical layer of enterocytes and their tight junction system. This type of local (gut) immunity consists of specialized cells and circulating chemicals shaped by the colonizing gut microbiome. For the maturation of the innate system, commensal microorganisms are necessary to distinguish between commensal and pathogenic bacteria, and immune cells are very fast in identifying and contrasting several foreign antigens [214]. The gut microbiome contributes to migration, the function of neutrophils, and division of T cells into types of T helper cells (Th), i.e., Th1, Th2, and Th17, or regulatory T cells [215,216,217]. Cells like Th17 (a subset of TCD4+ cells) secrete cytokines and regulate immune homeostasis and inflammation [218]. A mature immune system will prevent aberrant immune responses, unneeded chronic inflammation, and several diseases [219], since the close interaction between the microbiome and the host enterocytes is essential for the optimal functioning of intestinal antimicrobial proteins, epithelial cells, natural killer T cells, innate lymphoid cells, macrophages, interleukin(IL)-17-producing T cells, intestinal and peripheral regulatory T cells (Tregs), and immunoglobulin A (IgA) [220]. Notably, immune homeostasis can be modulated by antibiotic treatment of the gut microbiome [221,222]. The gut microbiome controls the production of antigen-presenting cells (APCs) which are ready to oppose the infection while retaining the immune tolerance to the normal gut microbiome [223]. Both intestinal and extraintestinal autoimmune diseases are associated with a dysregulated gut microbiome [224,225].

**Figure 3:**
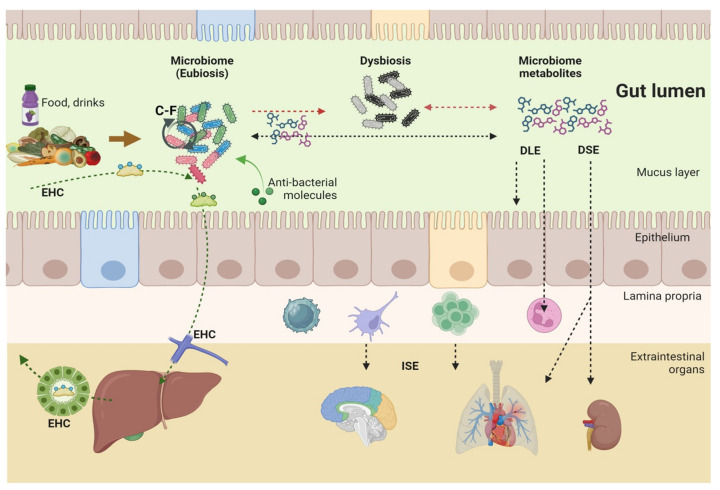
Effects of microbiome metabolites on the immune system and different targets. Food and nutrients encounter the microbiome highly represented in the colon. The microbiota metabolites serve as the nutrients for some bacteria and can shape the composition of the gut microbiome in health (eubiosis) and disease (dysbiosis). Bacterial cross-feeding (C-F) is when one bacterium is taken up by or exchanges its bacterial products with another microbe (e.g., lactate). Bacterial metabolites can act in different ways, i.e., by direct local effect (DLE) on either enterocytes and/or immune cells in the lamina propria. Such local effects can activate further systemic pathways. Microbiome metabolites can be absorbed and transported to remote organs to exert direct systemic effects (DSEs) or elicit indirect systemic effects (ISEs). Metabolites can also induce the host to release antibacterial molecules into the gut lumen. Enterohepatic circulation (EHC) is another example of the biotransformation of secreted primary bile acids to secondary bile acids by the resident colonic microbiome. Created with Biorender.com.

The gut microbiome has additional mechanisms to modulate both intestinal immune system development and intestinal inflammation, i.e., the polysaccharide A component of the resident intestinal microbe *Bacteroides fragilis* and SCFAs [226,227,228,229,230,231]. Microbial metabolites can act by a direct local effect on enterocytes and the immune system and activate further systemic pathways contributing to the maturation and development of the immune system (Figure 3).

## 6. Can Malnutrition and Fasting Influence the Gut Microbiome?

The gut microbiome function can be heavily shaped and re-shaped by lifestyles, including dietary habits as well as food supplements. An unhealthy, hypercaloric high-fat diet is a risk factor for overweight, obesity, metabolic syndrome, and diabetes [2,19,77,109,122,232,233]. Virtually all such metabolic abnormalities can be associated with dysbiosis. In addition, disrupted circadian rhythm is a predisposing condition to intestinal dysbiosis and metabolic and inflammatory disorders, including diabetes, intestinal inflammatory diseases, and cancer [234]. Due to its highly resilient features, the gut microbiome is responsive to malnutrition and fasting [235], as shown in animal models [236]. Further studies will be instructive in human models of malnutrition, such as in children who grow up with immature microbiomes, which keep changing during growth and with different diets. Malnourished children do not completely recover from malnourishment, likely because of an immature gut microbiome [237].

Intermittent fasting can induce weight, metabolic effects, and adipocyte status changes. The effects contribute to shape and optimize intestinal microbiome and intestinal immune responses [238,239]. In children with acute malnutrition, the malnutrition reappeared when the microbiome remained immature, underscoring the close relationship between food and the microbial community [240]. Diet, fasting, and time-restricted eating all influence several microbial products including SCFAs, trimethylamine N-oxide, tryptophan, and tyrosine derivatives suggesting that the gut microbiome might become a target of therapy to reduce malnutrition-dependent mortality.

## 7. How Is the Gut Microbiome Related to Major Human Diseases?

**Figure 4:**
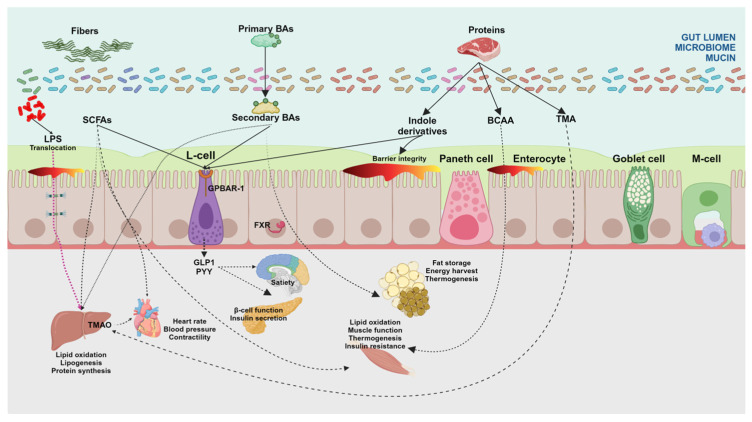
Principal modulatory effects of gut microbiome on host metabolism. The pleiotropic effect of microbiome-derived metabolites is evident on gluco-lipid and protein metabolisms, satiety, adipocyte functions (both white and brown adipose tissues involved in energy storage and non-shivering thermogenesis, respectively), muscle and heart function, and insulin sensitivity, among others. Short-chain fatty acids (SCFAs) such as acetate can inhibit lipolysis and increase adipogenesis. This effect is associated with the improved capacity of lipid storage of adipose tissues. SCFAs also influence pancreatic β-cell function and insulin secretion via the receptor GPR43, as well as lipid, carbohydrate, and protein metabolism of skeletal muscle through G protein-coupled receptors GPR41 and GPR43 and hydroxycarboxylic acid receptors (HDACs). The effect of SCFAs is seen also in the heart as an energy source and decreases heart rate, cardiac contractility, and blood pressure. Similarly, in the liver, SCFAs are an energy source; propionate is used for the synthesis of glucose and acetate can be used as substrates to synthesize cholesterol and long-chain fatty acids. Energy expenditure increases and hepatic steatosis decreases with SCFAs since hepatic lipogenesis is switched to hepatic beta-oxidation, a mechanism that contributes to protect against high-fat diet-induced obesity and metabolic dysfunction-associated steatotic liver disease (MASLD) [77,241]. Bile acids (BAs) in the terminal ileum target the membrane-associated G protein-coupled bile acid receptor 1 (GPBAR-1) with effects on the release of gut hormones such as glucagon-like peptide-1 (GLP-1) and peptide YY (PYY), involved in the regulation of appetite and gut motility. The interaction of BAs with the nuclear farnesoid X receptor (FXR) increases liver glycogen synthesis and insulin sensitivity, pancreas insulin secretion, and increased energy metabolism in the liver, brown adipose tissue, and muscles. Also in the liver, BAs contribute to regulating triglyceride metabolism, very low-density lipoprotein, and lipogenesis [57,83,143]. BCAAs contribute to protein synthesis, glucose and lipid metabolism, insulin resistance, hepatocyte proliferation, and thermogenesis of BAT. Other microbiome-derived metabolites are TMAO, tryptophan, and indole derivatives which, at different levels, are involved in energy and nutrient metabolism. If dysbiosis occurs, translocation of harmful metabolites such as lipopolysaccharides (LPSs) can occur across the leaky gut with further negative systemic effects. Legend: BAs, bile acids; BCAAs, branched-chain amino acids; GLP-1, glucagon-like peptide-1; LPSs, lipopolysaccharides; PYY, peptide YY; SCFAs, short-chain fatty acids; TMA, trimethyl amine; TMAO, trimethylamine-N-oxide [71]. Created with Biorender.com.

In eubiosis, the dominant, nonpathogenic gut microorganisms grow in specific niches and play a suppressive role in pathogenic colonization and growth. Major aspects that undergo crosstalk with the microbiome include digestion and metabolism, intestinal epithelial cell proliferation and differentiation, gut health, and brain–gut communication (Figure 4).

In general, several diseases are associated with low microbial diversity of the human microbiome [242], although population-specific comparisons of the microbiome are necessary between healthy and ill individuals, and when dysbiosis occurs, invasion by opportunistic pathogens will invade and colonize empty niches. At this stage, local changes in the intestinal barrier will result in increased gut permeability [19,49]. Sometimes pathogens are already resident, as in the case of *Clostridioides difficile* infection [243].

**Figure 5:**
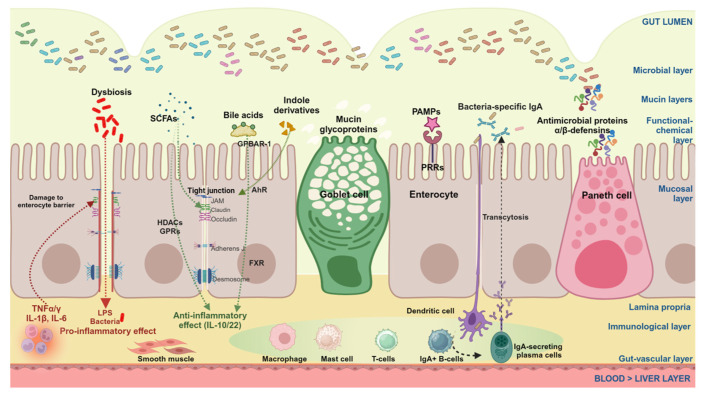
Crosstalk between microbiome and gut barrier. In the gut lumen, the complex morpho-functional multilayer gut barrier consists of several levels labeled on the right part of the figure. In health, microbes populate the central mucin layer, i.e., glycoproteins secreted by the epithelial Goblet cells. In eubiosis, microbes never come in close contact with the enterocytes. The functional–chemical layer is a combination of gut peristalsis and secretions of gastric acid, pancreatic juice, liver bile, antimicrobial proteins produced by the Paneth cells, and bacteria-specific immunoglobulin A (IgA) secreted by mature IgA-secreting plasma cells. The mucosa layer consists of epithelial specialized cells (here we show the enterocytes, the Goblet cell, and the Paneth cell). Intestinal epithelial cells present the pattern-recognition receptors (PRRs) able to sense the pathogen-associated molecular patterns (PAMPs) of microorganisms in the lumen. The lamina propria hosts the muscularis mucosa consisting of smooth muscle cells and the immunological layer, a complex and highly responsive set of macrophages, mast cells, and T- and B-lymphocytes which can be activated by dendritic cells presenting microbial derivatives and leading to the maturation of IgA-secreting plasma cells, which will secrete bacterial-specific IgA permeating the enterocyte by transcytosis. The gut vascular layer includes the endothelial cells. This level prevents the translocation of bacteria and/or microbial components across the extracellular and the intestinal epithelial barrier. A further layer is the liver barrier where resident macrophages (Kupffer cells) keep the liver free of bacteria. The set of structures that control the intercellular permeability between enterocytes includes the tight junctions, i.e., junctional adhesion molecules (JAMs, Claudin, occludin), the adherents junction, and the desmosome from the brush border to the basolateral membrane [245]. SCFAs, bile acids, and indole derivatives can enhance the physical barrier via increasing tight junction proteins. SCFAs can cross the barrier and interact with several targets such as histone deacetylases (HDACs) and G protein-coupled receptors (GPRs). Indole derivatives can interact with the aryl hydrocarbon receptors (AhRs) and can also protect the tight junction machinery. Bile acids can cross the enterocytes and produce effects on the nuclear farnesoid X receptor and the membrane-associated G protein-coupled bile acid receptor 1 (GPBAR-1). All such effects contribute to maintaining the immunological response in tune with the gut luminal events and can lead to the release of anti-inflammatory cytokines such as IL-10 and IL-22. In the presence of disease, (events are depicted on the right side of the slide as dysbiosis) there is closer contact between the microbiota and the enterocytes, followed by disruption of the junction system and translocation of lipopolysaccharides and/or bacteria. Thus, translocation triggers the activation of immune cells and leads to the production of pro-inflammatory cytokines which initiate the vicious cycle acting on local epithelial cells to worsen the physical barrier. Extraintestinal organs can be affected by the leaky gut condition, especially in the chronic state. Adapted from [49]. Created with Biorender.com.

The novel pathogenic microbiome is responsible for the production of dysregulated metabolites potentially harmful to the host. In addition, with the “leaky gut”, microbe-derived products can permeate the gut barrier, e.g., virulence factors, metabolites (e.g., lipopolysaccharide), and additional luminal components (Figure 5).

In this scenario, the normal function and crosstalk between the microbiome and gut barrier are disrupted, a condition contributing to aberrant immune-inflammatory responses. Potential consequences include inflammation, allergy, and autoimmune disorders mediated by molecular mimicry and abnormal T-cell response [244].

**Figure 6:**
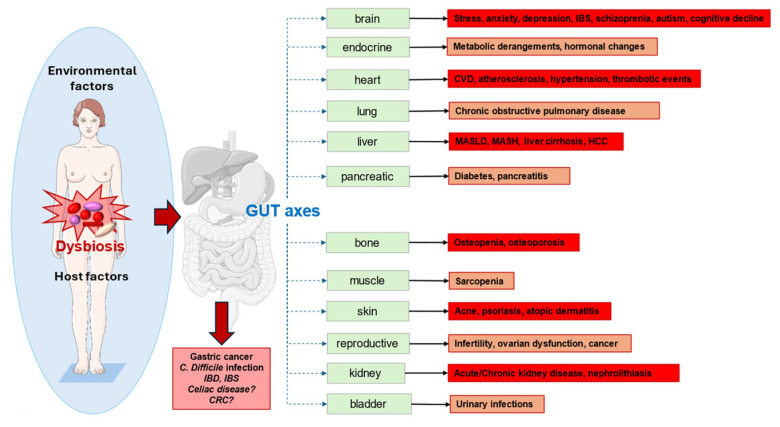
Potential clinical consequences of gut dysbiosis. With dysbiosis, the classical axes originating from the gut microbiota (green boxes) can be disrupted at various levels (red/orange boxes) and will contribute to the onset/perpetuation of disease. Local (gut) consequences are also shown. Abbreviations: CRC, colorectal cancer; CVD, cardiovascular disease; HCC, hepatocellular carcinoma; IBD, inflammatory bowel disease; IBS, irritable bowel disease; MASH, metabolic dysfunction-associated steatohepatitis; MASLD, metabolic dysfunction-associated steatotic liver disease. Created with https://smart.servier.com/.

The ultimate knowledge of pathophysiological mechanisms involved in the function of the gut microbiome in both health and disease is hindered by inter-individual variation [246]. Dysbiosis can originate from several conditions, including bacterial infections, dietary changes, aging, and the use of antibiotics [247]. Gut microbiome manipulation, e.g., bringing dysbiosis back to eubiosis, is becoming a potential strategy to influence the pathogenesis and expression of diseases [46,248,249,250], including obesity, diabetes, cardiovascular disorders, cancer, hypertension, and IBDs [46,251,252] (Figure 6).

Many diseases have been associated with various types of dysbiosis. Some of these conditions will be discussed in the following paragraphs, but more studies are needed to unravel the ultimate role of gut microbes in this respect. In general, in type 1 diabetes, genetically predisposed individuals develop an autoimmune response against pancreatic B-cells. Defects in microbiome development, decreased variability of microbial species, or dysbiosis may contribute to dysfunctional immunity with the devastation of autoimmune B-cells and increased leakiness of the intestinal epithelial barrier [253].

In asthma, abnormalities can imply outbreaks of *Chlamydophila pneumoniae* during bronchitis, and *pneumoniae* development affects the airway microbiome. The gut microbiome is influenced by the early life environmental introduction of the microbiome. In a pathological environment and response, the immune function growth and the development of defenses against allergic sensitization can be heavily affected [254]. Malnutrition can be associated with decreased or missing species that either process food categories efficiently or produce vitamins that may reduce the absorption of nutrients. An overabundance of *Enterobacteriaceae* can lead to epithelial damage, diarrhea, and limited absorption of nutrients [255]. Food-borne pathogens and food poisoning can imply a role for opportunistic pathogens (*Campylobacter*, *Salmonella*, *Escherichia coli*, *Shigella*, etc.) with the onset of gut dysbiosis [256]. *B. infantis* can have antidepressant effects. Depression can also involve a dysbiotic shift of *Bifidobacterium infantis*, generally found in infants’ gastrointestinal tracts and given as probiotic drugs [257]. Notably, oral administration of *Campylobacter jejuni* subclinical doses in murine models induced anxiety-like behavior without stimulating immunity. By contrast, in a marine model, *Lactobacillus* and *Bifidobacterium* may act with an anxiolytic influence [258].

Beyond their ability to regulate colitis, mouse models show that the gut microbiome can modulate the development of other diseases, namely metabolic syndromes, diabetes, and autism [259,260,261].

### 7.1. Obesity

Obesity accounts for millions of deaths and disability-adjusted life-years [262], and the prevalence of overweight and obesity is rising steadily worldwide. Obesity has nearly tripled since 1975, and in 2016, more than 1.9 billion adults (39%) were overweight, and over 650 million (13%) were obese [263], a total that is six times more than what was reported in the 1990s [264]. According to projections, by 2030, more than 1 billion people will live with obesity. Obesity is a multifactorial, chronic, relapsing, non-communicable disease characterized by an abnormal and/or excessive accumulation of body fat that presents a risk to health [265]. Obesity paves the way for a range of other non-communicable and communicable diseases [266,267,268]. Major causes of obesity remain behavioral changes with decreased energy expenditure [269], excess caloric intake [270], and environmental factors which influence the metabolic homeostasis [271,272,273]. Epigenetic modifications develop through multiple environment–gene interactions [273,274,275], with the involvement of the genetics of the molecules regulating energy homeostasis in the central nervous system [276,277].

The increasing rate of obesity goes along with other metabolic disorders, e.g., type 2 diabetes, and comorbidities which include cardiovascular diseases, metabolic syndromes, metabolic dysfunction-associated steatotic liver [278,279], cholesterol cholelithiasis [280], and different types of cancer [281,282,283]. Such comorbidities generate huge health costs in every national health system, irrespective of age.

Much attention is devoted to the management of obesity [284] and several studies are focusing on the role of the gut microbiome in obesity [15]. Studies in humans and mice show that changes in gut microbiome composition may play a vital role in the development of obesity [285,286,287,288]. For 8 weeks, 40 overweight or obese people were randomly assigned to diets high in protein and low in calories. The dietary intervention altered the microbial makeup and diversity, increasing the relative abundance of *Akkermansia* spp. and *Bifidobacterium* spp. while decreasing the enrichment of *Prevotella-9*, which is found to be high in obese people [289]. A type of obesogenic gut microbiome can contribute to obesity and include *Firmicutes*, *Bacteroidetes*, *Lactococcus*, *Rhizobium*, and *Clostridium* [290].

The positive correlation between the *Firmicutes*/*Bacteroidetes* ratio and obesity is confirmed [291]. One pathway might involve the hyperproduction of the microbiome-dependent SCFA butyrate, a substrate of extra energy for the host. Additional elements include low-grade inflammation due to intestinal microbiome metabolites [290], together with the activation of genetic background and epigenetic changes and microbiome-induced reduction of fatty acid oxidation due to the suppression of adenosine monophosphate kinase (AMPK) [292]. In muscle fibers and the liver, AMPK serves as a cellular energy sensor; its suppression leads to decreased fatty acid oxidation and increased fat accumulation. Chronic inflammation in relation to the microbiome can further explain several metabolic disturbances observed in obesity [293]. The gut microbial community can ferment dietary polysaccharides from fibers that are not digested by humans [294] and the production of SCFAs. SCFAs absorbed into the enterocyte can stimulate lipogenesis and increase triglyceride storage via molecular pathways. The fasting-induced adipocyte factor (FIAF), which inhibits lipoprotein lipase (LPL) and leads to triglyceride accumulation in adipocytes, can be suppressed by SCFAs [294].

The gut microbiome plays an essential causal and regulatory role in obesity. Clinical studies have demonstrated that shifts in certain bacterial populations, microbial byproducts, and microbial interactions can influence the development of obesity. An imbalance in the microbiome, along with decreased microbial diversity, is associated with obesity. Specifically, lower microbial diversity and a reduction in *Bacteroides* species are frequently observed in individuals with obesity [295,296].

### 7.2. Diabetes Mellitus

Type 2 diabetes mellitus (T2DM) is a heterogeneous disease that consists of hyperglycemia, insulin resistance, and relative impairment in insulin secretion. The ongoing status of insulin resistance plays a role in the genesis of other T2DM abnormalities which include inflammation, lipoprotein abnormalities, hypertension, and several other metabolic abnormalities. The common disorder is T2DM and its prevalence increases steadily with increasing degrees of obesity [297] and with age. The prevalence of T2DM has risen alarmingly in the past decade [298,299] and this trend is partly due to a parallel rise in obesity and a sedentary lifestyle [300].

There were roughly 463 million people globally with diabetes in 2019, and this number is supposed to increase to 700 million by 2045 [299]. The gut–intestinal microbiome appears to be involved in the evolution of T2DM [13,301], due to the strong interplay of diet, caloric intake, obesity, gut microbiome, and the onset of T2DM [302]. In this respect, a dietary shift from a low-fat, plant polysaccharide-rich diet to a high-fat, high-sugar diet is sufficient to modify the microbiome composition rapidly [303]. Over the years, the dietary shift was from fibers to fats and this is a reason for microbial adaptation, along with metabolic disorders [2,304]. Compared to controls, the new-onset type 1 diabetes subjects develop a distinctive gut microbiome with lactate- and butyrate-producing bacteria assembling mucin and maintaining gut integrity in the control group. In type 1 DM, by contrast, mucin synthesis was inhibited by non-butyrate-producing lactate-utilizing bacteria, a condition that contributes to the autoimmunity of β-cells and type [305]. Other studies show changes in gut microbiome profiles in type 1 DM; increased occurrence of *A. muciniphila* is inversely related to the probability of developing type 1 DM [306,307], and *A. muciniphila* could become a potential probiotic in the treatment of type 1 diabetes. The ultimate knowledge of mechanisms linking gut bacteria to the development of diabetes certainly requires additional studies [308,309,310]. Concerning T2DM, mechanisms likely involve the interplay between the microbiome and changes in butyrate and incretin secretions [311,312]. A moderate degree of intestinal dysbiosis can occur in T2DM, with decreased occurrence of butyrate-producing bacteria, along with increased opportunistic pathogens [312]. Additional pathways can be influenced and include glucose homeostasis, insulin signaling, and inflammation [138,312].

In humans, fecal microbiota transplantation (FMT) from lean, healthy donors to obese individuals has been shown to improve insulin sensitivity, suggesting that reshaping the gut microbiome can directly benefit metabolic functions [313]. In addition, insulin resistance in T2DM has been linked to increased circulating levels of branched-chain amino acids (BCAAs), influenced by specific gut microbiota. This relationship is thought to exacerbate insulin resistance, illustrating how microbial metabolites directly impact metabolic pathways associated with T2DM [314]. *Akkermansia muciniphila*, a mucin-degrading bacterium, is associated with improved metabolic health. Studies show that increased abundance of *A. muciniphila* improves glucose tolerance, reduces systemic inflammation, and enhances lipid metabolism in obese patients [315]. The positive effects of *A. muciniphila* are partly attributed to its outer membrane protein, which interacts with Toll-like receptor 2, enhancing gut barrier function and reducing inflammation [316].

### 7.3. Hypertension

Hypertension is an important risk factor for cardiac, stroke, and kidney diseases [317]. About 1.56 billion individuals worldwide will suffer from hypertension by 2025 [252]. Both genetic and environmental factors contribute to hypertension, and the latter include excessive dietary salt intake, sedentary life, and alcohol consumption [318,319]. Gut dysbiosis appears to influence hypertension [320,321] and the microbiome could be soon incorporated into novel antihypertensive therapies. The ratio of *Bacteroidetes* and *Firmicutes* is associated with hypertension [322], since hypertensive animals and hypertensive patients had an abundance of gut *Bacteroidetes* and *Firmicutes*, as sequenced by 16S ribosomal RNA [323]. Experiments in germ-free mice infused with angiotensin II-infused suggest that the gut microbiome, via angiotensin II, is likely involved in vascular dysfunction and hypertension [324]. In addition, SCFAs derived from the gut microbiome can modulate blood pressure [325]. The action of SCFAs on G protein-coupled receptor (GPR) pathways reflects on renin secretion and blood pressure [326]. A by-product of intestinal microbial fermentation, lyxose, was higher in patients with newly diagnosed hypertension compared to healthy controls [327]. In accord with this evidence, *Lactobacillus* can bring beneficial effects in the regulation of blood pressure [328]. Nevertheless, more studies are needed in this field.

### 7.4. Cardiovascular Diseases (CVDs)

The incidence of CVDs is increasing worldwide and also in low- and middle-income countries [329]. In CVD, a possibility is the decreased perfusion of the gut, a condition leading to gut barrier dysfunction [2,19]. Eubiosis is essential for the maintenance of the physiological function and selective permeability of this barrier [2,19,57,330,331]. The gut microbiome might take part in the pathogenesis of heart disease and stroke [332,333,334]. Indeed, wrong dietary habits, lifestyles, and toxic chemicals contaminating ingested food or water will predispose individuals to the altered diversity and relative abundance of the gut microbiome with disrupted gut barrier function, intestinal immune system, and secretion of local peptides and Ig with antimicrobial function [122]. In patients with CVDs, a correlation exists between the amount of fecal gut microbiome and the increase in intestinal permeability [335]. In addition, patients positive for serum bacterial DNA had higher plasma levels of inflammatory markers, i.e., highly sensitive C-reactive protein and interleukin-6 levels, than patients without bacterial DNA in peripheral blood [336]. The abundance of *Streptococcus* and *Enterobacteriaceae* is increased and linked with coronary artery disease [337]. Patients with coronary artery disease have a type of dysbiosis, i.e., decreased *Bacteroidetes* and increased *Firmicutes*. Notably, TMAO is a small molecule metabolite originating from the bacterial fermentation of ingested dietary choline and carnitine contained in fish, eggs, and red meat. In atherosclerosis, TMAO plays an important role and can help predict cardiovascular risk [145,338,339,340,341,342,343]. Atherosclerotic CVDs have been associated with bacterial genes coding for trimethylamine (TMA) lyase [344], while TMA lyase might generate TMAO, a step recalling the function of the gut microbiome in relation to dietary choline and carnitine biotransformation gut microbiome [345]. Thus, TMAO is deemed as a specific microbiome-derived factor contributing to atherosclerotic CVD [346,347].

### 7.5. Cancer

Globally, cancer is the second most common cause of death [348], with several risk factors involving the combination of exposure to pathogens, pollutants, UV radiation, lifestyles, dietary habits, and toxic substances on a genetic background [123]. The gut microbiome can also play a role in cancer development at different levels [349]. The gut microbiome can also be involved in cancer risk reduction and tumor growth, and be the target for anti-cancer therapies, as shown by metabolomics and metagenomics studies [350]. Studies in germ-free mice also suggest that gut microbiome plays a role in the development and progression of cancer [351].

In patients with prostate cancer, studies found a greater abundance of *Bacteroides massiliensis* and less abundance of *Eubacterium rectale* and *F. prausnitzii*, as examples of microbes involved in the pathogenesis of this cancer [352].

In gastric cancer, the role of *H. pylori* infection cannot be neglected, since *H. pylori* becomes the predominant bacterium in the stomach of *H. pylori*-infected patients [53], and this bacterium can disrupt the gastric environment, which also includes the resident, non-pathogenic microbiome [52]. In this scenario, the risk of gastric cancer increases [353], while *H. Pylori* eradication will reverse gastric dysbiosis and increase microbial diversity again [354]. Further studies are needed to unravel the complex mechanisms involved in gastric cancerogenesis and dysbiosis.

Important changes in the gut microbiome can contribute to the development of colorectal carcinoma in genetic and carcinogenic tumorigenesis models [123,351]. In colorectal cancer, the occurrence of *Fusobacterium nucleatum*, *Bacteroides fragilis*, and *Peptostreptococcus anaerobic* has been reported [355]. In addition, *F. nucleatum* and *Clostridium colicanis* might act as markers of carcinogenesis [356]. Notably, *F. nucleatum* can suppress the immune response of the host and promote cellular proliferation. In line with these microbial changes, a diet enriched in whole grains and dietary fiber yields a lower risk of *F. nucleatum*-positive cancer. Dietary changes such as a high-fat diet can disrupt the mucin composition and microbiome populations and density with further carcinogenic potential in the colon [357,358]. This finding indicates that the gut microbiome is bridging dietary habits with colorectal cancer development. Fungi and viruses can also impact the gut microbiota and the host [359]. For example, a study identified signature fungi in colorectal cancer and adenoma.

Patients from multiple cohorts observed trans-kingdom interactions between enteric fungi and bacteria in colorectal cancer progression [360].

Recent research highlights the critical role of gut microbiota in influencing the efficacy of cancer immunotherapy, particularly immune checkpoint inhibitors like anti-programmed cell death protein 1 (PD-1) therapy. Studies show that cancer patients with a more diverse gut microbiota respond better to anti-PD-1 therapies than those with a less diverse microbiota. Specifically, responders’ microbiota are enriched with bacterial taxa commonly linked to health, including *Akkermansia*, *Faecalibacterium*, and *Bifidobacterium*, all of which are often depleted in non-responders [361,362,363].

Experimental evidence further supports this link. In mouse models, transplanting fecal microbiota from responders (patients who showed a favorable response to anti-PD-1 therapy) led to improved antitumor T-cell responses and enhanced efficacy of the therapy [362]. Additionally, supplementing non-responder microbiota with specific bacterial taxa enriched in responders, such as *Akkermansia muciniphila*, boosted the antitumor response to PD-1 blockade in these animals, underscoring the potential therapeutic effects of certain beneficial microbes [363].

These findings suggest that gut microbiota profiling could potentially serve as a predictive biomarker for immunotherapy responsiveness. Patients with a microbiota profile associated with higher diversity and specific beneficial taxa might be more likely to benefit from therapies like anti-PD-1.

Also in this area, further studies are required to better understand the role of the gut microbiome in cancer development and the role of novel anti-carcinogenic strategies by targeting the gut microbiome.

### 7.6. Inflammatory Bowel Disease (IBD)

Chronic inflammatory gut pathological conditions, such as ulcerative colitis (UC) and Crohn’s disease (CD), are encompassed within IBD. Both diseases are characterized by diarrhea, rectal bleeding, abdominal pain, fatigue, and weight loss, but they differ in clinical manifestations of inflammation and intestinal localization [364]. A chronic condition characterized by relapsing and remitting episodes of inflammation limited to the mucosal layer of the colon is UC, while CD is distinguished by transmural inflammation with intervals of normal, disease-free areas. The highest prevalence in Western countries is for IBD, and the incidence in newly industrialized countries has risen rapidly (Africa, the Middle East, Asia, and South America) [365]. In many respects, IBD is a type of gut microbiome-associated disease characterized by dysbiosis [366], reduced species richness and diversity, and lower temporal stability [367]. Changes in environmental factors involving antibiotic use, metabolic abnormalities, dietary changes, etc., have likely contributed to the increased prevalence of IBD during the past century. In early childhood, the increased use of antibiotics increases the likelihood of developing IBD [368,369,370,371]. Depending on the studies, certain microbial taxa including bacteria, archaea, fungi, and viruses are enriched or depleted in IBD, but results differ between studies [372]. Gut dysbiosis in IBD patients may affect the homeostasis of fatty acids, amino acids derivatives, and BAs [373], and such changes can become another important gateway to oxidative stress, inflammatory pathways, and nutritional dysregulation [374,375,376,377,378,379], with changes appearing as early as childhood, but little is known about the ultimate role of microbial alterations as primary drivers of IBD or secondary to the underlying IBD. Communities of bacteria from UC patients induce Th17 responses when transferred into mice [380,381]. This experiment points to the important immunological modulatory role of T cells by the microbiome suggesting that changes in communities of intestinal bacteria contribute to the initiation and/or perpetuation of IBD inflammation.

The intestinal lumen can host microbiome and microbial-derived factors that may promote IBD on top of an underlying genetic immune defect. In IBD, the gut microbiome likely acts as an important multifunctional inflammatory factor. Studies suggest that intestinal microbiome diversity decreases in IBD patients [382,383]. Pathogenic bacteria in IBD belong to the phylum *Proteobacteria* [384] which can initiate chronic inflammation [385]. The abundance of *Ruminococcus gnavus* is also found to be higher in IBD [386], a potential marker of increased oxidative stress [387]. Decreased diversity of phyla *Proteobacteria* and *Firmicutes* occur in IBD patients. The *Firmicutes* species include *Clostridium leptum*, *F. prausnitzii* [366,388], and the butyric-acid-producing *Roseburia* spp. [389]. Also, a decreased *Firmicutes/Bacteroidetes* ratio was observed in IBD patients, including CD, compared to the healthy microbiome [375]. Disrupted gut microbiome and lower *Firmicutes* occurred in patients with active UC or aggressive CD [390]. Following the onset of dysbiosis, IBD patients are prone to develop aberrant mucosal immune responses, along with defective butyrate production, a primary energy substrate for colonocytes [389,391]. The host can initiate immune responses directed against microbial components, such as antibodies to the DNA segment (I2). This finding occurs in the affected mucosa of 54% of CD patients compared with 4–10% of controls [392]. Complicated CD patients show antibodies to bacterial flagellin [393,394]. More aggressive IBD correlates with immunoreactivity to microbial antigens [395,396]. Increased antibodies likely depend on enhanced exposure to intestinal bacteria due to epithelial barrier disruption and/or to increased inflammatory response of intestinal immune cells.

Antibodies further contribute to the pathogenesis of IBD. The role of the gut microbiome can involve the protection, induction, and/or maintenance of disease in murine IBD models. A normal microbiome is necessary in rodent models of colitis and intestinal inflammation but not in germ-free conditions [396,397,398]. Bacteria from mice with colitis promote intestinal inflammation when transferred from one animal to another [115,116], and an underlying genetic defect can further promote the inflammatory response [399]. IgA-coated intestinal bacteria preferentially drive intestinal inflammation [400]. The protective effect is seen with certain combinations of bacteria (e.g., probiotics, *Clostridia*) which can mediate protection from inflammation through such mechanisms as inducing Treg cells [401] or modulating growth factors able to promote epithelial healing [402].

Research strongly supports the role of gut microbiota in the causation and progression of IBD. Key findings from clinical trials suggest several mechanisms by which microbiota impact IBD. For example, CD and UC patients show a loss of bacterial diversity, with notable decreases in beneficial bacteria such as *Faecalibacterium prausnitzii* [403]. Conversely, there is an expansion of pro-inflammatory bacterial families like *Enterobacteriaceae*, whose overgrowth is associated with new-onset CD [379]. In addition, microbial products in UC patients have been shown to influence immune cell differentiation, increasing the Th2/Th1 ratio in T cells. Severe UC cases often involve expansions of *Bacteroides* and *Candida*, which correlate with pro-inflammatory immune responses and more intense disease symptoms. This highlights the link between microbial composition, immune response profiles, and disease severity [404].

A potential therapeutic strategy in IBD patients can be the amelioration of immunological homeostasis by modifying the gut microbiome [405]. Nutrition and dietary management for adults with inflammatory bowel disease can also include fiber intake because of a beneficial effect on commensal gut bacteria. Indeed, a high intake of dietary fiber, mainly from fruit and cruciferous vegetables, appears to decrease the risk of CD but not UC [406,407]. Some dietary fibers are substrates to form SCFAs, which stimulate water and sodium absorption in the colon and can promote mucosal healing [408].

Probiotics are living nonpathogenic microorganisms. When ingested, probiotics can have a positive influence on host health and physiology. For UC patients, some probiotics have shown promising results, e.g., *E. coli* Nissle 1917 and VSL#3. Preparations validated for clinical use are still missing. Available data do not support the clinical effectiveness of probiotic therapy for either induction or maintenance of remission in CD patients. The use of probiotics in the management of patients with IBD, including those with pouchitis, is discussed separately. Nevertheless, the knowledge about the ultimate role of the gut microbiome as a contributing factor to IBD progression requires additional studies.

### 7.7. Irritable Bowel Syndrome (IBS) and Celiac Disease

Several studies suggest that IBS patients often undergo types of dysbiosis with either an increase or decrease in bacterial density along with qualitative/relative changes [409]. Patients with IBS, as compared to healthy controls, can show a significant reduction in the concentration of *Lactobacillus* species within the *Firmicutes* phyla [410], as well as an increased ratio of phyla *Firmicutes* to *Bacteroidetes* [411,412,413] and decreased density of genera *Lactobacilli* and *Faecalibacterium* (phyla *Firmicutes*), as well as genera *Bifidobacteria* and *Collinsella* (phyla *Actinobacteria*). Studies also show that IBS patients can develop an abundance of genera *Veillonella*, *Streptococci*, *Ruminococcus* spp. (phyla *Firmicutes*), and *Enterobacteriaceae* spp. (phyla *Proteobacteria*). Altogether, a large variability of findings exists in IBS patients, likely reflecting study type, population characteristics, laboratory methodologies, and clinical expression of disease. Nevertheless, the above-mentioned findings point to the disruption of healthy microbiota and change in epithelial barrier function in IBS patients [412,413].

Among the *Actinobacteria* phylum, the *Bifidobacterium* genera counts were lower in celiac disease [414]. In most celiac disease patients, there is an increase in the number of Gram-negative bacteria, such as *Bacteroides*, *E. coli*, and *Enterobacteriaceae*, while the number of Gram-positive bacteria, including *Bifidobacterium*, *Streptococcus*, and *Lactobacillus* spp., is decreased compared to healthy individuals [415].

Gut bacteria maintain a balanced condition (eubiosis) to aid digestion, immunological response, and pathogen defense. Dysbiosis compromises the intestinal barrier, resulting in inflammation and immunological diseases. This is associated with disorders such as diabetes, asthma, malnutrition, and depression. Restoring eubiosis by microbiota modification is being investigated as a treatment for obesity, cardiovascular disease, and autoimmune illnesses. Microbiome research is underway to better understand its role in health and illness. The gut microbiome, which includes bacteria such as *Firmicutes* and *Bacteroidetes*, plays an important role in obesity by altering metabolic pathways and causing low-grade inflammation. Type 2 diabetes mellitus is associated with gut dysbiosis, since high fat and sugar diets disturb gut flora, causing inflammation and affecting glucose management. This dysbiosis is analogous to the microbial imbalances associated with hypertension and cardiovascular disease. Gut dysbiosis also increases cancer risk and development, with certain bacteria linked to certain malignancies. IBD such as CD and UC are frequently associated with considerable microbial imbalance, underlining the gut’s involvement in immune regulation and inflammation.

## 8. Any Novel Strategy for Engineering the Microbiome to Target Different Diseases?

So far, the main intervention strategies able to modulate the gut microbiome include diet/nutrition, dietary supplements, drugs, and fecal microbiota transplantation (FMT). Engineering the microbiome to achieve various therapeutic outcomes in the host is feasible [4]. Both in vitro and in vivo assessments of different methods to modulate the microbiome composition and function show promising results.

Potential therapeutic applications in the topics discussed show a wide range of applications.

Probiotics have been employed in obesity and diabetes, with the rationale that *A. muciniphila* is depleted in patients with obesity, type 2 diabetes, and hypertension [416]. The novelty of *A. muciniphila* lies in its potential as a next-generation probiotic. Research suggests that restoring this beneficial microbe may help improve metabolic health and enhance gut barrier function, offering a promising avenue for therapeutic interventions in these conditions [417]. Obese patients who received the probiotic (pasteurized *A. muciniphila* with better efficacy than live bacteria) for three months showed improved insulin sensitivity and reduced cholesterol levels. In IBD, *F. prausnitzii* abundance is decreased [418]. The probiotic reduced disease severity in a mouse model of colitis [419]. Clinical trials have been difficult to design since bacteria are difficult to cultivate. *Saccharomyces boulardii* could be used as an adjuvant to induce remission or prevent the relapse of IBD, but clinical trials with the probiotic alone are lacking [420].

Prebiotics, such as FOS, have been used in CD patients who showed improvement in disease symptoms and increased fecal *Bifidobacteria* [421]. In colorectal cancer, the developed colon-retentive inulin gel increased the abundance of beneficial *Bifidobacteria* and *Akkermansia*. This goal enhanced the antitumor efficacy of immune checkpoint blockers [422].

Targeted antibiotics could become an option for *Clostridioides difficile* infection. Ridinilazole is a small DNA-binding molecule that specifically targets *C. difficile*. In a Phase 2 clinical trial, ridinilazole was superior to standard-of-care vancomycin [423].

Bacteriophages have therapeutic potential in colorectal cancer. *F. nucleatum* causes chemoresistance in colorectal cancer, but in a mouse model, a phage isolated from saliva and used in conjunction with a chemotherapy drug was more effective than chemotherapy alone [424].

Enzyme inhibitors have been used in colon cancer to inhibit the bacteria β-glucuronidase and to prevent the conversion of a safe byproduct of the anticancer drug into toxic SN-38. The tested enzyme inhibitors did not harm the bacteria and mammalian cells [425]. In CVDs, the protocol of inhibition of TMA lyases was used, since microbial TMA lyases are involved in TMAO synthesis, which is associated with CVDs. In mouse models, the tested inhibitors were safe for microbes and their use is associated with TMAO decrease [426,427].

Engineered microbes have been tested in diabetes and obesity. The engineered *Bacillus subtilis* was metabolically rewired to produce 1.5 g/L butyrate in vitro and in an obesity mouse model, the microbe retarded weight gain and fat accumulation [428]. In IBD, a genetically engineered *S. cerevisiae* using a human P2Y2 receptor could sense extracellular ATP (eATP) and produce ATP-degrading enzymes. In a colitis mouse model, the engineered yeast *S. cerevisiae* was better than standard-of-care IBD therapies.

Natural microbial consortia are currently used for recurrent *C. difficile* infection in fecal microbiota transplantation, where fecal samples from healthy donors are administered to patients. The risk of pathogen transmission must be considered, together with the rate of success, depending on the genetics and lifestyle of the recipient [429]. Another approach might consider the Oral administration of Bacterial Spores from Healthy Donors (SER-109), a procedure characterized by a reduced risk of pathogen transmission, as seen in the Phase 3 trial [430].

The use of synthetic microbial consortia might play a role in *L. monocytogenes* infection and cancer. The consortium of 11 bacterial strains which have low abundance in the gut, obtained from healthy donors, can promote CD8+ T cells in the intestine. This effect likely enhances the efficacy of immune checkpoint inhibitors in tumor models [431]. In human IBD, GUT-108, i.e., a consortium of 11 bacterial strains can mimic some reduced functions such as the production of antimicrobial factors to prevent pathogen growth and the induction of anti-inflammatory molecules [432].

Nevertheless, all therapeutic approaches, but fecal microbiota transplantation, require further convincing and successful clinical translation evidence.

## 9. Conclusions

The human gut microbiome is an ecosystem carrying a diverse set of genomes and acting as the second human genome. After colonization by the environmental mother’s vaginal and fecal microbiomes during birth, microbial life is in symbiosis with the host throughout its life. The gut microbiome plays a crucial role in human health through microbiome−host interactions which involve pathways of digestion, metabolism, immunity, and brain function. By contrast, gut dysbiosis can be a contributing factor to several diseases acting as a driver of inflammatory, metabolic, cancer, and immunological abnormalities.

The interest in the potential modulation of the microbiome and the use of probiotics to prevent dysbiosis or to cure gut microbial abnormalities is gaining popularity within the scientific community, given the fact that the gut microbiome has been implicated in several diseases including obesity, cardiovascular diseases, hypertension, cancer, inflammatory bowel disease, and several others. Nevertheless, the causal relationship and molecular mechanisms linking environmental factors, microbial communities, and the onset and development of disease remain largely unclear in most studies. Further well-designed clinical trials will help to better understand how the microbiome can interact with the host, and the complex interplays with several possible external confounders.

Eubiosis and dysbiosis represent the continuously fluctuating aspect of health and disease and the mechanisms linking the gut microbiome with the host will pave the way to therapeutic and preventive strategies. This aspect is important when considering the need to fight obesity and cancer. A multilevel, multidisciplinary “one-health” approach is ideal in this area of research and clinical development.

The many drawbacks of microbiome investigations include the fact that big and complicated data sets need training for optimal analysis, which are not often available globally, correlating with the necessity for the representation of biological samples. Low- and middle-income nations, which have most of the human microbial diversity and health concerns, require large investments in bioinformatics training so that scientists may easily use and create projects incorporating sequence data. Open research, particularly meta-analyses of previously released microbiome data, can lead to new theories and knowledge [433].

## Figures and Tables

**Table 1:** Principal factors influencing abundance and diversity of the human microbiome.

Intrinsic Factors	Extrinsic Factors
Genetic background Nature of body environments Ethnicity Gender Age	Mode of delivery during birth Breastfeeding vs. formula milk feeding Dietary habits Physical activity Smoking Immune activity Medications Toxic chemicals Geographic location Air pollution Climate Seasonality
